# High vulnerability of medial prefrontal pyramidal neurons in post‐stroke, vascular, Alzheimer's disease, and aging‐related dementias

**DOI:** 10.1002/alz.71151

**Published:** 2026-02-12

**Authors:** Dan D. Jobson, Yoshiki Hase, Lauren Walker, Tuomo Polvikoski, Ahmad A. Khundakar, Louise Allan, Raj N. Kalaria

**Affiliations:** ^1^ Faculty of Medical Sciences, Translational and Clinical Research Institute Newcastle University, Campus for Ageing & Vitality (Health Innovation Neighbourhood) Newcastle upon Tyne UK; ^2^ School of Health and Life Sciences Teesside University Middlesbrough UK; ^3^ National Horizons Centre Teesside University Darlington UK; ^4^ College of Medicine and Health University of Exeter Exeter UK

**Keywords:** Alzheimer's disease, frontotemporal dementia, mitochondria, morphometric, post‐stroke dementia, stereology, vascular dementia

## Abstract

**INTRODUCTION:**

The medial prefrontal cortex (mPFC) is critical for executive function, behavioral inhibition, and memory. Its high vulnerability to dementia, compared to other prefrontal regions, remains unclear.

**METHODS:**

We analyzed *post mortem* brain tissue from 118 older subjects, including post‐stroke survivors, Alzheimer's disease; vascular, mixed, and frontotemporal dementia (FTD); and cognitively unimpaired controls. Three‐dimensional stereology was used to assess pyramidal neuron densities and volumes in mPFC layers III and V. Immunohistochemistry evaluated metabolic dysfunction via cytochrome *c* oxidase subunit 1 (COX1), cytochrome *c* oxidase subunit 4 (COX4), and 78 kDa glucose‐regulated protein expression.

**RESULTS:**

Pyramidal neuron densities were lowered by ≈ 45% and volumes by ≈ 37% within all dementia groups relative to controls, except for FTD densities. COX1 and COX4 mitochondrial markers were consistently reduced across dementias. Neuronal densities declined with age, especially in the sixth decade of life. Other prefrontal areas were less affected.

**DISCUSSION:**

The mPFC shows high neuronal vulnerability in dementia, while suggesting a vascular–metabolic mechanism, with implications for targeted therapeutic strategies.

**Highlights:**

- Severe pyramidal neuron loss and atrophy arose in the medial prefrontal cortex.
- Neuronal morphometric changes correlated with cognitive status or aging effects.
- Metabolic changes decreased by the greatest extent in vascular‐associated dementias.
- Metabolic neuronal markers correlated with aging and frontal vascular pathology.

## 1 BACKGROUND

Post‐stroke dementia (PSD) is defined according to established guidelines as immediate or delayed irreversible cognitive decline within 6 months after a stroke event.^1^ An array of heterogeneous cognitive deficits remains remarkably high, with ≈ 40% of post‐stroke survivors at 1 year exhibiting signs of cognitive impairment.^2^, ^3^ This causes a significant burden for mortality and disability, which is expected to increase exponentially by 60% given projected rises in stroke incidence by 2035.^4^, ^5^, ^6^ We previously reported in the longitudinal Newcastle Cognitive Function After Stroke (CogFAST) study that ≈ 1 in 4 post‐ischemic stroke survivors developed clinical features of dementia and would survive for a median age of ≈ 6.72 years.^7^ Stroke events may further bring forward dementia diagnosis by ≈ 10 years, which can effectively diminish cognitive reserve and lead to a stepwise decline to dementia.^8^, ^9^ Intriguingly, nearly 80% of PSD cases fulfil diagnostic criteria for vascular dementia (VaD), with features of cerebral small vessel disease and thus represent a subtype of VaD, which rather encompasses a broader scope of individuals possessing clinical dementia symptoms at baseline due to extensive vascular pathology without necessarily the occurrence of an acute stroke event.^1^, ^7^ However, deciphering the crucial neuropathological hallmarks for why a post‐stroke survivor may develop dementia remains elusive.

Extensive evidence suggests that frontal lobe–associated executive dysfunction is a key feature of cerebrovascular disease–associated dementias, particularly when assessing the effects of lesions to critical frontal–subcortical circuits.^10^, ^11^ Numerous neuroimaging studies have also indicated disruption in subcortical connectivity from the medial aspects of the prefrontal cortex (mPFC) across diverse aging‐related dementias.^12^ Lesions resulting from vascular damage and degeneration within frontal‐subcortical circuits are thought to reflect neuronal loss and executive dysfunction associated with VaD.^11^, ^13^ We have previously shown that a specific vulnerability of glutamatergic pyramidal neurons occurs in the dorsolateral prefrontal cortex (dlPFC). Neuronal volumes were lower by 30% to 40% within cortical layers III and V across various dementias, including PSD, Alzheimer's disease (AD), VaD, and mixed (AD/VaD) dementia, compared to post‐stroke non‐dementia (PSND) and age‐matched control subjects. The neuronal volume changes further correlated to measures of cognitive dysfunction and clinical dementia ratings, without significant changes in neuronal densities or neurodegenerative pathology. These observations implicated a vascular basis for the highly localized neuronal atrophy evident in all common dementias, including PSD and VaD.^14^

A positron emission tomography neuroimaging study recently reported that severe synaptic loss occurs within both medial and dorsolateral frontal areas of frontotemporal dementia (FTD) subjects, yet the mPFC remains understudied.^15^ We therefore determined the status of mPFC pyramidal neurons in layers III and V, in relation to clinical variables such as cognitive status, vascular pathology, and aging effects. Our recent transcriptomic profiling study intriguingly suggested numerous disparate gene expression patterns within pyramidal neurons in PSD subjects relative to age‐matched controls. One common pathway reported as significantly changed within PSD cases was metabolic functioning, specifically involving impaired mitochondrial energy production, as reported within chronic hypoperfusive areas leading to cellular dysfunction.^16^, ^17^

In this study, we investigated the mPFC in dementia disorders, including FTD, in view of its extensive neurodegenerative pathology manifesting in the frontal lobes linked to executive dysfunction, which could therefore potentially display the most severe neuronal morphometric changes. We also analyzed pyramidal neurons immunolocalized by three metabolic markers, namely cytochrome *c* oxidase subunit 1 or 4 (COX1 or COX4) and 78 kDa glucose‐regulated protein/binding immunoglobulin protein (GRP78/BiP). COX1 and COX4 were selected as reliable markers of mitochondrial oxidative capacities, which have revealed reduced subunit expression and activity in the frontal cortex of senile dementia and inherited mitochondrial diseases,^18^, ^19^, ^20^, ^21^ whereas GRP78 has previously exhibited several neuroprotective roles within endoplasmic reticulum–stress response pathways, by guarding against oxidative stress and glutamate excitotoxicity due to prolonged cerebral ischemia.^17^, ^22^, ^23^, ^24^ Given our previous review^12^ we tested the hypothesis that mPFC pyramidal neurons substantially degenerate across the common aging‐associated dementias, which could enable precise therapeutic targeting.

**RESEARCH IN CONTEXT**

1. **Systematic review**: The authors reviewed the relevant literature from PubMed and Scopus online databases. While pyramidal neurons have previously shown prefrontal sub‐region–specific morphometric changes across numerous aging‐associated dementias, which neuroimaging studies report as connectivity aberrations associated with cognitive decline, the precise changes within the medial prefrontal cortex remain unclear.
2. **Interpretation**: Our evidence indicates medial prefrontal pyramidal neurons display widespread reductions in neuronal soma densities and volumes across varying dementias, including Alzheimer's disease, and post‐stroke and vascular dementias. The study further implicated mitochondrial dysfunction within neurons due to reduced metabolic marker expression across all dementia groups, but particularly in vascular‐related dementias.
3. **Future directions**: Our findings suggest a high vulnerability for medial prefrontal pyramidal neurons driven predominantly by vascular lesions in the absence of neurodegenerative pathology, which may instigate neuronal metabolic dysfunction. This may provide a novel early‐stage biomarker to track neurodegenerative change as well as a clinically relevant therapeutic target.

## 2 METHODS

### 2.1 Study design and total subject demographics

Frontal lobe brain tissue was acquired from the Newcastle Brain Tissue Resource (NBTR) and analyzed for a total of 118 subjects (Table 1). The cohort included 15 FTD cases, which were diagnosed (53%) with progressive supranuclear palsy (PSP), as well as corticobasal degeneration, Pick's disease, and autosomal dominant four‐repeat tauopathy with behavioral variant FTD. The study cohort included 25 post‐ischemic stroke survivors from the Newcastle CogFAST longitudinal study, separated into those with or without a diagnosis of dementia according to Diagnostic and Statistical Manual of Mental Disorders, Fourth Edition criteria.^7^, ^14^

**TABLE 1 alz71151-tbl-0001:** Details of total subject demographics and pathological features.

Variable	YC	OC	PSND	PSD	VaD	Mixed	AD	FTD
No. of subjects (*n* = 118)	10	22	13	12	17	14	15	15
Age, years (range)	71.5^d^ (64–87)	85.9 (71–99)	84.2 (78–89)	87.1 (80–98)	82.7 (70–97)	83.6 (72–94)	83.9 (68–96)	76.6 (62–93)
PMD, hours (range)	48.5 (9–93)	35.8 (12–105)	38.9 (11–76)	38.6 (17–88)	48.5 (14–84)	36.4 (9–69)	42.9 (5–94)	53.2 (10–216)
Braak (range)	1.40 (0–3)	2.00 (0–3)	2.39 (0–5)	2.67 (0–4)	2.00 (1–4)	4.75^d^ (2–6)	5.39^d^ (3–6)	2.30 (2–3)
CERAD (range)	0.00 (0)	0.18 (0–1)	1.46 (0–2)	1.17 (0–3)	0.75 (0–2)	2.50^d^ (0–3)	3.00^d^ (3)	0.40 (0–2)
Alzheimer's Disease Neuropathologic Changes; A, B, C (mean)^a^	A0.0, B0.5, C0.0	A0.5, B1.2, C0.5	A0.5, B1.2, C0.7	A0.5, B1.2, C0.8	A0.6, B1.2, C0.8	A2.5, B2.6, C2.6	A3.0, B3.0, C3.0	A1.3, B1.7, C0.3
Vascular pathology scores (range)^b^	NPD	NPD	12.5 (7–14)	13.2 (9–17)	14.3 (12–15)	11.7 (8–14)	5.10 (4–7)	13.3 (9–18)
VCING ratings^c^	Low	Low	High	High	High	High	Moderate	High
WM pathology scores (SEM)	NPD	NPD	2.92 (0.08)	2.83 (0.17)	3.00 (0)	2.67 (0.33)	1.13 (0.09)	2.50 (0.17)

*Notes*: Mean group values are displayed for the total subjects (*n*) analyzed, followed by the range in parentheses. Subjects from each disorder group were respectively separated into either the stereology or densitometry analyses, with at least 10 cases per group. The most frequent causes of death in subjects included cardiac arrest, bronchopneumonia, and carcinoma, with no significant variation in prevalence between the disorder groups. The *post mortem* tissue fixation time ranged from 3 to 44 weeks for all cases, with no clear differences for potential tissue shrinkage across all groups. Total vascular and white matter pathology scores were further quantified using LFB and H&E immunostained diagnostic slides for each case using established criteria described previously.

Abbreviations: AD, Alzheimer's disease; CERAD, Consortium to Establish a Registry for Alzheimer's Disease; FTD, frontotemporal dementia; H&E, hematoxylin and eosin; LFB, Luxol fast blue; Mixed, mixed Alzheimer's disease and vascular dementia; No., number; NPD, no pathological diagnosis; OC, older controls; PMD, *post mortem* delay; PSD, post‐stroke dementia; PSND, post‐stroke non‐dementia; SEM, standard error of the mean; VaD, vascular dementia; WM, white matter; YC, younger controls.

^a^ Alzheimer's Disease Neuropathologic Changes.^36^

^b^ Vascular Pathology Scores.^35^

^c^ Vascular Cognitive Impairment Neuropathology Guidelines (VCING) ratings.^37^

^d^ Significance: indicates differences were found between group means with the OC (*p* < 0.05).

First‐time hospitalized ischemic stroke patients aged ≥ 75 years old were enrolled into the longitudinal study if deemed as not having dementia 3 months post‐stroke and had no disabilities preventing the completion of neuropsychological cognitive assessments (Table 2). A standardized battery comprising medical history, Mini‐Mental State Examination (MMSE), Cambridge Cognition Examination (CAMCOG), neurological deficit assessment, blood screen, and computed tomography neuroimaging review was undertaken at the time of the stroke event and then repeated at annual intervals.^7^ Stroke was defined according to the World Health Organization's definition and classified by the Oxford Community Stroke Project (OCSP) for all ischemic stroke patients assessed within this study, which further presented with remote lesions and no ischemic areas localized to the frontal cortex, as evidenced by the precise stroke lesion location and size data for each[Table alz71151-tbl-0001] subject (Table 2).^25^

**TABLE 2:** Clinical features of the post‐stroke and vascular dementia subjects.

Variable (region)		PSND	PSD	VaD
Time from first stroke (months)	Mean (± 2 SE)	81.3 (35.9)	63.3 (13.4)	N/A
Time from baseline to death (months)	Mean (± 2 SE)	65.1 (15.9)	64.6 (12.8)	Dementia
Total CAMCOG score (/100)	Mean (range)	89.8 (83–99)	60.8 (24–80)	43.5 (0–64)
Total MMSE score (/30)	Mean (SEM)	27.4 (0.40)	16.4 (1.49)	<16
Total memory sub‐score (/27)	Mean (2SEM)	22.5 (1.17)	14.8 (2.62)	<14
Total executive function score (/28)	Mean (SEM)	16.9 (0.82)	9.5 (1.19)	<9
Clinical Dementia Rating	Mean (SEM)	0.00 (0.00)	1.35 (0.24)	3.00 (0.00)
Hemisphere with visible change or not ^a^	None, left, right, both	2, 2, 3, 3^c^	2, 5, 3, 1^c^	N/A
OCSP stroke classification^b^	LACS, PACS, TACS, POCS	5, 2, 1, 2^*^	4, 4, 3, 1	N/A
Location of stroke lesions (%)^d^	Cerebral cortex, WM, BG/Thalamus, Cerebellum/Brainstem	33, 22, 31, 14	25, 21, 33, 21	28, 22, 30, 20
Size of stroke lesions (%)^d^	<5 mm, 5–15 mm, 16–30 mm, 31–50 mm	82, 12, 4, 2	87, 11, 0, 2	88, 10, 1, 1

*Note*: Values show mean (SEM or range) unless otherwise stated.

Abbreviations: BG, basal ganglia; CAMCOG, Cambridge Cognition Examination; CT, computed tomography; LACS, lacunar stroke; MMSE, Mini‐Mental State Examination; mPFC, medial prefrontal cortex; N/A, not applicable; OCSP, Oxfordshire Community Stroke Project; PACS, partial anterior circulation stroke; POCS, posterior circulation stroke; PSD, post‐stroke dementia; PSND, post‐stroke non‐dementia; SE, standard error; SEM, standard error of the mean; TACS, total anterior circulation stroke; VaD, vascular dementia; WM, white matter.

^a^ Visualized upon a CT scan.

^b^ OCSP Classification: there were no differences found between stroke territory distributions of post‐stroke with no dementia and post‐stroke dementia cases (*p* > 0.05).

^c^ Only eight cases were included for post‐stroke non‐dementia hemisphere and OCSP classification, as three of the analyzed cases had unknown or not classifiable criteria, whereas one additional post‐stroke dementia case's lesion location was unknown upon CT imaging.

^d^ Data on individual cases were extracted from Hase et al.^23^ None of the cortical or subcortical (WM) lesions were within the region of interest in sections, that is, the mPFC in the analysis.

All brain tissues diagnosed as VaD, mixed dementia, AD, or FTD were retrieved from subjects included in our prospective memory clinic studies.^26^ Aging control subjects were obtained as NBTR referrals, with no diagnoses of any neurological or psychological illness or cognitive impairment, which were further separated into either younger (YC) or older (OC) controls. Neuropathological assessment using established diagnostic criteria was completed to provide a final diagnosis of all subjects with dementia, as previously described.^7^, ^27^ All further morphological analyses were performed under blinded operator conditions to prevent bias, with samples identified by using coded sequential numbers. Ethical approvals were granted by local research ethics committees for this study (Newcastle upon Tyne Foundation Hospitals Trust), along with consent permission according to the Declaration of Helsinki for *post mortem* brain tissue research from next‐of‐kin or family.

### 2.2 Stereological analysis

**FIGURE 1 alz71151-fig-0001:**
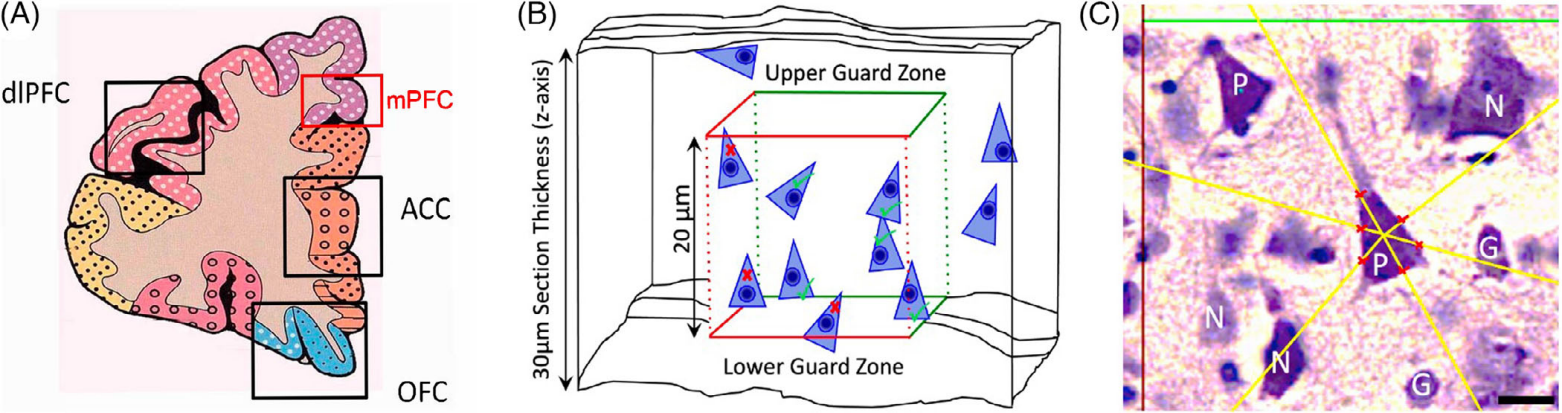
Medial prefrontal region, stereological analysis, and the nucleator probe principle diagrams. A, Neurons from the dorsal mPFC region (red box) were sampled per the coronal atlas of the brain.^12^, ^26^ B, A 20 µm box depicts the counting frame for pyramidal neurons, with a 5 µm upper or lower guard zone that removes potential tissue artifacts. The stereological method only counted neurons at randomly located sites within the frame or touching the green inclusion lines (green checkmarks).^30^ C, Cresyl fast violet‐stained pyramidal neuron (P) with six yellow rays radiating from its center at the nucleolus. The nucleator probe lines intersect with the cell perimeter boundary (red crosses) to enable calculation of individual soma volume using the Stereo Investigator software. G or N letters, respectively, denote glial or non‐pyramidal cells; red and green lines delineate the optical disector counting box frame. Scale bar = 20 µm. ACC, anterior cingulate cortex; dlPFC, dorsolateral prefrontal cortex; mPFC, medial prefrontal cortex; OFC, orbitofrontal cortex.

Formalin‐fixed paraffin‐embedded (FFPE) coronal blocks containing no visible infarcts or lesions were selected to include the dorsomedial cortical aspects of Brodmann area nine, according to the Newcastle brain map (Figure 1A).^28^ Three cresyl fast violet‐stained tissue sections of 30 µm thickness for each case were analyzed using standardized protocols in triplicate by three‐dimensional stereology, as identical tissue blocks and sections used previously by Foster et al.^14^, ^29^, ^30^, ^31^ Images were captured on a Leica DM6 B fluorescence motorized microscope, with a 10MP CMOS camera connected to a computer.

Stereo Investigator (MBF Bioscience) version 2019.1.1 software was used to collect the morphometric data from at least 10 subjects per disorder group, which considered six regions of interest at randomly selected sites of the same area for each case's mPFC gyri (Figure 1A), with > 240 slides analyzed in total for our Z‐stacking to generate robust results. A 5 µm guard zone volume at the top and bottom of each section removed potential artifacts arising from the mechanical cutting of tissue sections. This left a 20 µm thick area for analysis (Figure 1B), which was still sufficient for minimizing any tissue shrinkage discrepancies.^32^, ^33^ Eight to twelve sites were then further sampled at 20× magnification within each region of interest for either cortical layer III or V, which were distinguished by the clustering of larger pyramidal neurons and accounted for > 4800 frames of analysis in total, as in prior prefrontal stereology studies.^14^, ^34^ Neuronal densities (in cells/mm^3^) were measured from raw cell counts obtained within an optical disector box, and overall densities were then calculated using the following equation:^35^

$$\mathrm{Nv}=\frac{\sum^{p^{-}}Q^{-}}{P\cdot V}$$

where: *Nv* = numerical density, *p* ^−^ = disector samples, *Q* ^−^ = number of objects counted, *p* = total number of dissectors, and *V* = disector box volume, in µm^3^ (0.250 × 0.250 × 0.020).

Neurons were selectively identified as in previous studies,^34^, ^36^ by displaying a single visible nucleolus, pale nucleus, and Nissl‐stained cytoplasm; unlike the spherical glial cells, without cytoplasmic Nissl staining or heterogeneous chromatin arrangement in the nucleus, to validate the positive count of neurons (Figure 1C). Approximately 176 neurons were analyzed for each cortical layer and case to obtain the neuronal density and volume measurements, which gave an appropriately low sampling error and neuronal volume coefficient of error of 0.0250, which was consistent with prior stereological recommendations.^14^, ^33^, ^37^

Individual neuronal volumes (in µm^3^) were estimated using the nucleator method,^38^ whereby a nucleator probe is positioned centrally within a nucleolus‐containing cell. Six randomly oriented lines are then fired outward with marks made where these probe lines intersect with the cell perimeter boundaries. From this information, Stereo Investigator can calculate an estimate of neuronal soma volume (Figure 1C). Resulting neuronal densities and volumes were quantified in triplicate by using three sections per case, and mean values (± standard error of the mean) per disorder group were calculated. The reliability in identifying and measuring neuronal morphometrics was ensured by reassessing randomly selected cases under blinded conditions. We established > 90% agreement between cases assessed at the start with those at the end of the stereological analysis period.

### 2.3 Cortical thickness assessments

Mean cortical thickness[Fig alz71151-fig-0001] was determined in adjacent prefrontal areas, accounting for the extent of mPFC tissue shrinkage.^14^ Briefly, measurements were taken from the cortical pial surface to the superficial white matter border and followed the general direction of neurons. This distance was drawn via the straight‐line tool on ImageJ2 (Fiji) version 2.16.0 software, with three brightfield images captured per case from each Nissl‐stained section. Three measurements were taken at the smallest visually observable distance at a gray matter sulcus and then averaged, to ensure consistency and prevent variations in the tissue sectioning angle.

### 2.4 Immunohistochemical and immunofluorescence analyses

FFPE frontal tissue blocks were serially cut into 10 µm sections containing the mPFC (Figure 1). After dewaxing, they underwent heat‐mediated antigen retrieval in citrate buffer (pH 6.0) solution in the microwave for ≈ 10 minutes, before being quenched for 20 minutes with 3% hydrogen peroxide and Tris‐buffered saline (0.1% Triton‐X100). Sections were blocked with 10% normal goat serum for 1 hour and then incubated overnight at 4°C with the primary antibody. For immunohistochemical staining, sections were incubated in either AT8 (1:1000, Innogenetics, Autogen Bioclear), GRP78/BiP (1:500, ab32618, Abcam), COX1/NDUFB8 (1:200, ab110242, Abcam), or COX4 (1:200, ab110261, Abcam) antibodies. The MACH4 Universal Horseradish Peroxidase (HRP)‐Polymer detection system (Biocare Medical) was then used, by adding the universal probe and then HRP‐polymer solutions for 30 minutes each. After the final wash, immunocomplexes were detected with 3,3′‐diaminobenzidine (DAB) and lightly counterstained in hematoxylin.

To reveal the extent of colocalizations by visual inspection between the neuronal metabolic markers, fluorescent immunolocalization was performed in adjacent mPFC sections. After undergoing identical dewaxing, blocking, and antigen retrieval steps, sections were incubated overnight at 4°C with the prior primary antibodies plus COX1 rabbit monoclonal (ab192878, Abcam) diluted at 1:100. After several wash steps in phosphate‐buffered saline, sections were incubated with respective Alexa Fluor 488 and 594 goat anti‐rabbit or anti‐mouse secondary antibodies (1:200, Invitrogen, Thermo Fisher Scientific) at room temperature for 2 hours. Finally, sections were washed in phosphate‐buffered saline, covered briefly in 0.3% Sudan Black B solution to quench background lipofuscin autofluorescence, and mounted in ProLong Mounting Media with DAPI (Invitrogen, Thermo Fisher Scientific) for positive cell nuclei detection. Five images were subsequently captured for each case and cortical layer, with the number of individual neurons colocalized (yellow signal) calculated as a percentage of total neuronal counts within each image, which enabled further calculation of mean frequencies of colocalized neurons.

### 2.5 Quantitative densitometric analysis

Quantitative densitometric analysis was undertaken to determine the per area percentage (p/a) and number of pyramidal cells stained by each of the COX1, COX4, and GRP78 markers. Within each randomly placed frame, we counted all clearly visible large pyramidal cells of triangular shape with a nucleolus. At least five brightfield images were captured at 20× magnification for each cortical layer and case, as preliminary analysis suggested that five images were sufficient to reliably estimate the cell numbers (*p* > 0.05; Figure S1 in supporting information). Mean area values were quantified via the wand tracing tool at a consistent tolerance setting in ImageJ2 (Fiji) version 2.16.0 software.

### 2.6 Statistical analysis

All statistical analyses were performed using Prism (GraphPad) version 10.2 with the alpha level of significance set at *p* ≤ 0.05. The Shapiro–Wilk test initially assessed the normal distribution of values. Data found not to be normally distributed were analyzed using non‐parametric statistical methods. Disorder group means were compared using one‐way analysis of variance with Tukey multiple comparisons test for normalized data or Kruskal–Wallis with Dunn multiple comparisons test for non‐normally distributed values. Either Pearson ρ (rho) correlation coefficient or Spearman rank was used to assess associations with parametric or non‐parametric clinical and neuropsychometric data, respectively.

## 3 RESULTS

### 3.1 Cohort clinical and neuropathological features

The mean age (years) was not different across all disorder groups and the OC group. The YC group was younger by a mean age of 14 years relative to OC subjects (*p* = 0.002; Table 1). There was no difference (*p* > 0.05) between the post‐stroke groups for the mean time from the first stroke and time from baseline to death (both in months), indicating dementia incidence and cognitive impairment separated the two post‐stroke groups. We additionally noted only minor differences among stroke territory distribution according to OCSP classification, such as more anterior strokes occurring within PSD, and most stroke events (36%) were classified as lacunar strokes (Table 2). All strokes were characteristic of cerebral small vessel disease. Ischemic stroke lesions were located remotely from the consistently normal appearing mPFC. Mean total scores of vascular pathologies also assessed the extent of arteriolosclerosis, perivascular space dilation, microinfarcts, and cerebral amyloid angiopathy, which were similar between post‐stroke and VaD subjects (Table 1).^39^

There were no differences in neurofibrillary tau pathology with Braak staging, neuritic plaque burden with Consortium to Establish a Registry for Alzheimer's Disease (CERAD), ABC scores, total vascular pathology scores, or Vascular Cognitive Impairment Neuropathology Guidelines ratings between the post‐stroke subjects.^40^, ^41^ As expected, Braak staging and CERAD scores were significantly greater for mixed dementia and AD cases, whereas AD had lower vascular pathology scores (Table 1). Assessment of neurofibrillary tangle burden further revealed negligible AT8 immunoreactivity in PSND, PSD, and VaD groups. The mixed dementia, AD, or FTD groups exhibited substantially greater hyperphosphorylated tau burden upon visual inspection (Figure S2 in supporting information), which was consistent with prior neurodegenerative pathology findings in other prefrontal sub‐regions.^14^

### 3.2 Neuronal densities in the mPFC

**FIGURE 2:**
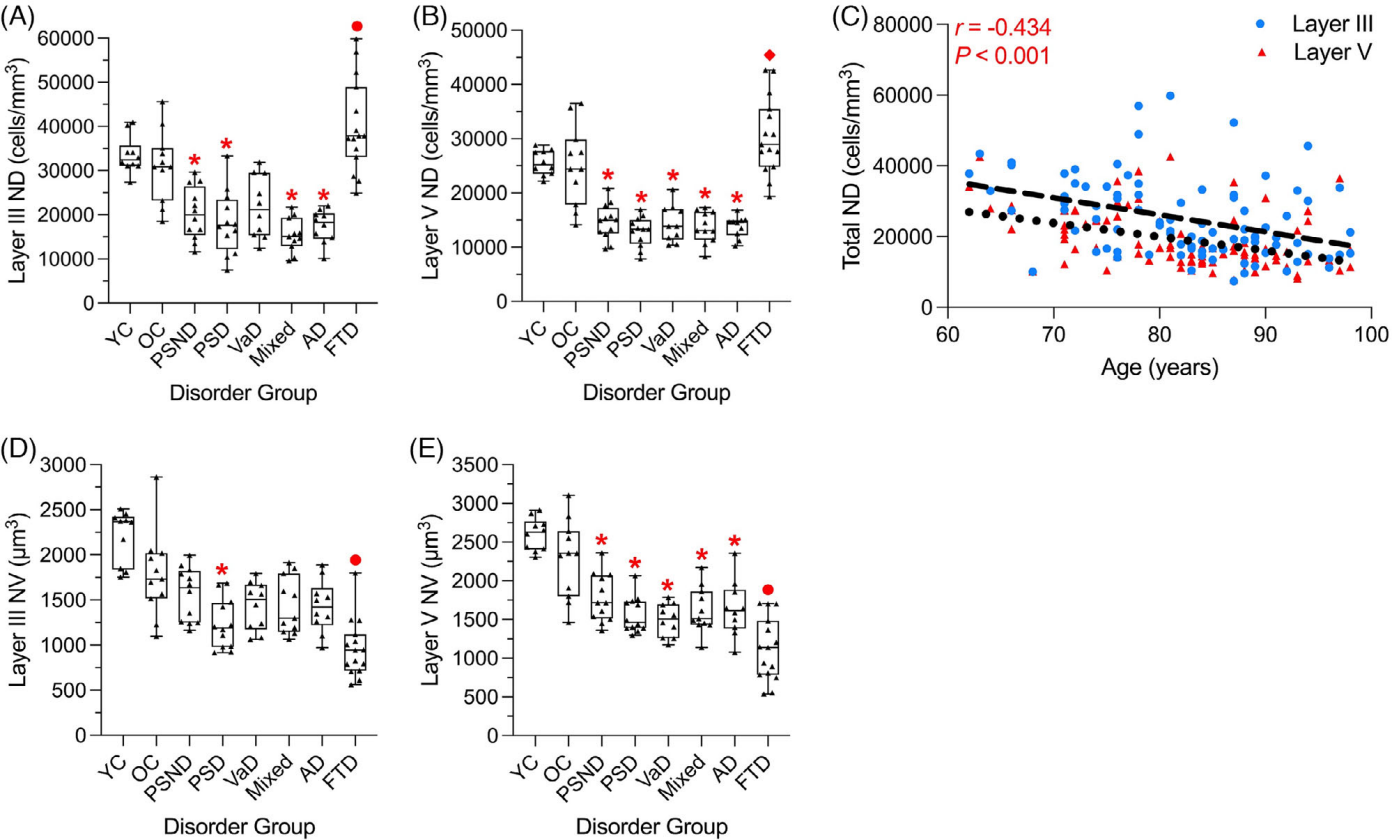
Neuronal densities and volumes of the mPFC pyramidal neurons in dementia and aging. Box plots show the interquartile range extending to minimum and maximum values for pyramidal neuron densities (cells per mm^3^) in layer III (A) and layer V (B), as well as pyramidal neuron volumes (µm^3^) in layer III (D) and layer V (E). FTD subjects exhibited higher neuronal densities, but also displayed the lowest volumes. C, Total neuronal densities across both layers III and V as a function of age of all subjects. The dashed lines signify 95% confidence limits of the linear regression line, while dashed or dotted lines‐of‐best‐fit specify either layer III or layer V. Spearman rank correlation coefficient was used due to the non‐parametric data. * significantly different mean values versus OC, ⬥ significant differences versus only PSND subjects, and • significant differences versus both age‐matched control and PSND cases (*p* ≤ 0.05). Raw data points are displayed as triangles. AD, Alzheimer's disease; FTD, frontotemporal dementia; Mixed, mixed dementia; mPFC, medial prefrontal cortex; ND, neuronal densities; NV, neuronal volumes; OC, older controls; PSD, post‐stroke dementia; PSND, post‐stroke non‐dementia; VaD, vascular dementia; YC, younger controls.

The estimated mean (and median) mPFC neuronal densities in layer III (25279 ± 1159 cells/mm^3^ in total) were consistently found to be greater than in layer V (19259 ± 854.7 cells/mm^3^ in total) across all disorder groups (Table S1 in supporting information). Neuronal densities in both layers III and V were lower in nearly all dementia groups by ≈ 45%, including PSD (*p* < 0.001), mixed dementia (*p* < 0.001), and AD (*p* < 0.001) subjects compared to the OC group (Figure 2). Neuronal densities were also reduced for VaD cases in layer V relative to OC (*p* < 0.001; Figure 2B). We further noted densities were reduced in the PSND group (*p* ≤ 0.009) with no apparent statistical differences for both cortical layers between the PSND and PSD subjects (*p* > 0.05; Figure 2). However, FTD subjects unexpectedly showed higher neuronal densities in both layers III and V (Figure 2; Table S1). We estimated the mean neuronal densities in FTD were observed to be greater in layer III, by ≈ 6000 to 9000 cells, compared to the OC and YC groups (*p* < 0.05).

In further analysis of the whole cohort, we found neuronal densities in both neocortical layers to be lower with increasing age (*r* = −0.434, *p* < 0.001; Figure 2C). We also calculated that in both layers III and V, neuronal cell densities were lower, most markedly in the sixth decade (60–69 years), but to a much lesser extent in the latter decades. By measuring the regression line slope (Figure S3 in supporting information), we found cells were being lost at a gradient of −1090.2 or −1051.9 on average in layers III or V, respectively. Although the individuals aged 60 to 69 years did possess the slight caveat of the smallest group size, this finding was still further emphasized by exhibiting an overall negative association within layer V (*r* = −0.851, *p* = 0.015; Figure S3A).

### 3.3 Neuronal volumes in the medial prefrontal cortex

We found layer V pyramidal neurons to be consistently larger compared to layer III neurons (Table S1). In the OC group, mean neuronal volume was 1778 µm^3^ in layer III relative to 2278 µm^3^ in layer V (*p* = 0.04). The mPFC neuronal volumes were also substantially affected by dementia, with a ≈ 37% significant lowering across both cortical layers compared to age‐matched control subjects (*p* < 0.001). However, volume reductions in layer III were significant only in PSD compared to OC (*p* = 0.003; Figure 2D). In contrast, there were widespread volume reductions in layer V relative to OC for all dementia groups (*p* < 0.001) and PSND subjects (*p* = 0.01; Figure 2E).

Furthermore, we observed extensive reductions in FTD subjects in both cortical layers. In layer III, FTD neuronal volumes were lower compared to YC, OC (both *p* < 0.001), or PSND (*p* = 0.006) plus VaD, mixed dementia, or AD subjects (*p* ≤ 0.01; Figure 2D). While in layer V, volumes in FTD subjects were reduced relative to YC, OC, or PSND (*p* < 0.001) as well as PSD (*p* = 0.02), mixed dementia, and AD cases (both *p* = 0.006; Figure 2E). Neuronal volumes in FTD, along with PSD and VaD, were reduced by the greatest extent (at ≈ 41%) across both cortical layers compared to controls. We also explored aging effects in neuronal volumes (Table S1). The OC exhibited smaller volumes overall by 12% to 19% compared to YC subjects, although these differences did not reach statistical significance for either cortical layer (*p* ≥ 0.36). However, only within combined YC and OC subjects, layer III neuronal volumes were found to be lower with increasing age (*r* = −0.648; *p* = 0.002) and not for neuronal volumes across total cases (*r* = −0.073; *p* > 0.05).

### 3.4 Cortical thickness and interlaminar neuronal comparisons

**FIGURE 3:**
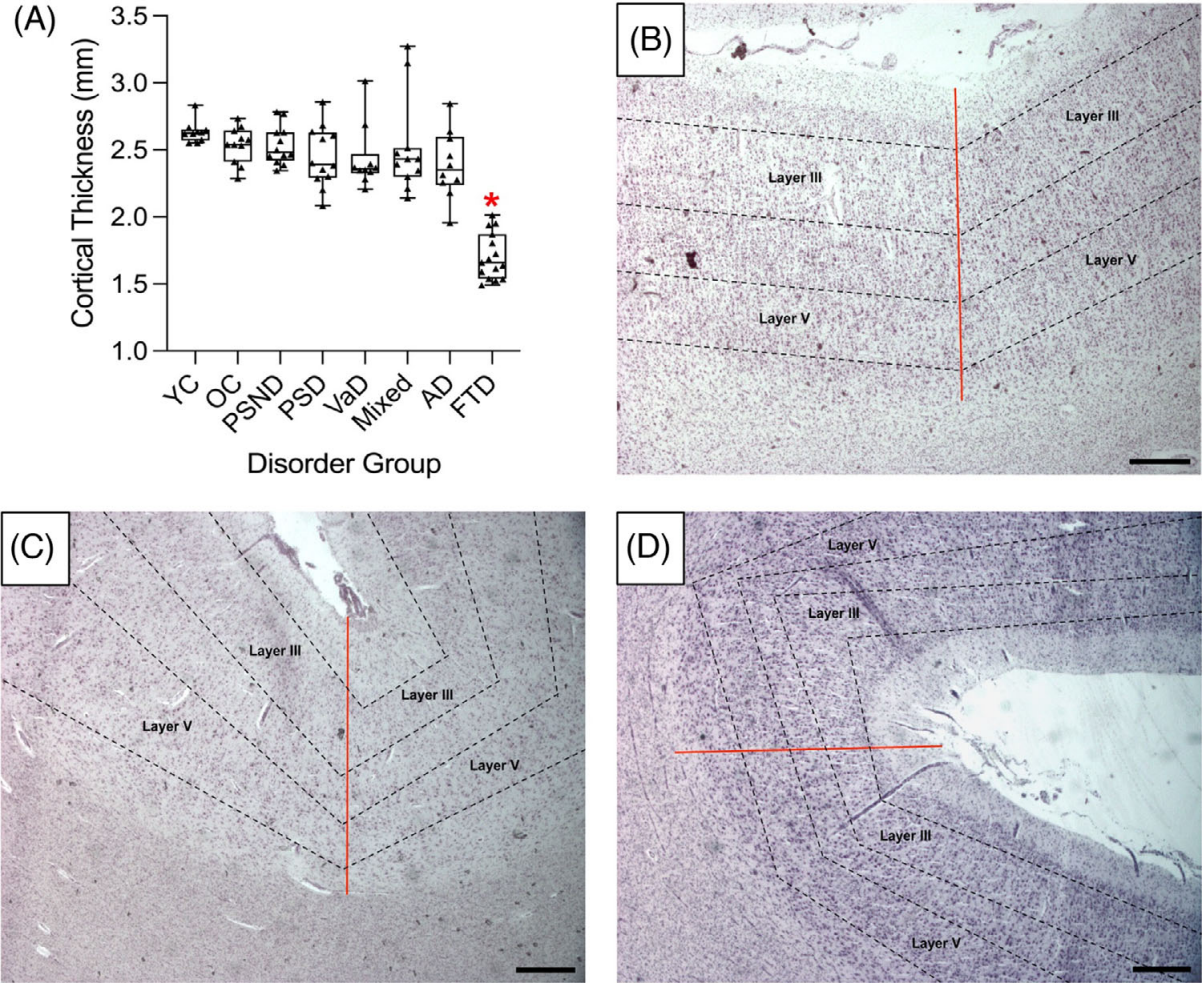
Morphological cortical thickness estimations to detect tissue atrophy. A, The box plot represents the interquartile range extending to the minimum and maximum values as mean mPFC thickness (mm) for 10 to 15 cases. This was quantified for all disorder groups and indicated disease‐specific tissue atrophy for FTD. Raw data points are illustrated as triangles. Representative images of cortical thickness measurements (straight red lines) at gray matter sulci to the white matter border are shown in YC (B), OC (C), and FTD (D) cases. Either cortical layer III or V is further delineated by black dotted lines. Scale bar = 500 µm. AD, Alzheimer's disease; FTD, frontotemporal dementia; Mixed, mixed dementia; mPFC, medial prefrontal cortex; OC, older controls; PSD, post‐stroke dementia; PSND, post‐stroke non‐dementia; VaD, vascular dementia; YC, younger controls.

We were concerned whether diffuse mPFC neocortical ribbon thinning was a potential factor, particularly in FTD cases exhibiting high neuronal densities and low volumes. We found that the mean cortical thickness was reduced in FTD cases (1.705 mm ± 0.044) relative to YC (2.632 mm ± 0.025) and OC (2.535 mm ± 0.040; *p* < 0.001; Figures 3A–D). There were no differences in cortical thickness within FTD, whether they were characterized as familial or sporadic four‐repeat tauopathy cases (*p* > 0.05; Figure S4 in supporting information). There was also no evidence that the length of *post mortem* interval or tissue fixation artifact was a factor, as the cases were routinely processed using the same protocols (Table 1). No further differences were noted for potential cortical thickness variation between all assessed dementia groups or age‐matched controls (Figure 3A), which corroborated previous neocortical atrophy findings and was within the expected mPFC thickness range of 2.4 to 2.8 mm.^14^, ^42^

We made further interlaminar comparisons of neuronal densities or volumes in layers III and V (Table S2 in supporting information). A consistent positive correlation of mPFC neuronal densities between layers III and V was found for all dementias, indicating that the widespread neuronal loss compared to OC may be unrelated to layer‐specific changes. Alternatively, for neuronal volumes, we elucidated that positive interlaminar correlations were not significant in cases with predominant vascular pathology, such as PSND, PSD, and VaD, yet positive associations were observed in mixed dementia (*p* = 0.008), AD (*p* = 0.037), and FTD (*p* < 0.001). These results reveal that neuronal atrophy may not arise similarly across cortical layers in different age‐related disorders.

### 3.5 Neuronal morphology in relation to cognition

**FIGURE 4:**
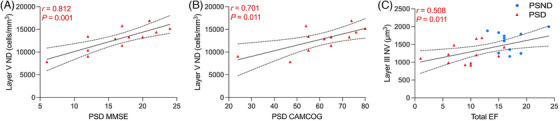
Neuronal measure associations with variables of cognitive function. Correlation curves show relationships between layer V densities relative to MMSE (A) and CAMCOG (B) scores in PSD subjects. C, Relationship between total executive function scores and layer III neuronal volumes in PS subjects. Pearson correlation coefficient was implemented due to the parametric data. CAMCOG, Cambridge Cognition Examination; EF, executive function; MMSE, Mini‐Mental State Examination; ND, neuronal densities; NV, neuronal volumes; PS, post‐stroke; PSD, post‐stroke dementia; PSND, post‐stroke non‐dementia.

Post‐stroke neuronal volumes in layer III were positively correlated with total MMSE (*r* = 0.653, *p* < 0.001) and CAMCOG (*r* = 0.618, *p* = 0.001) measures of cognitive impairment. We also found that neuronal densities in layer V positively correlated with MMSE (*r* = 0.812, *p* = 0.001) and CAMCOG scores (*r* = 0.701, *p* = 0.011) in PSD cases (Figures 4A,B). There were also positive associations further demonstrated for total executive function scores with layer III neuronal volumes (*r* = 0.508, *p* = 0.011) in post‐stroke subjects (Figure 4C), therefore suggestive that those with greater mPFC neuronal loss or atrophy display poor cognitive status. No significant associations for the type of ischemic stroke according to OCSP classification were additionally noted for neuronal densities or volumes (*p* > 0.05).

### 3.6 Densitometric analysis of the metabolic neuronal markers

To confirm the stereological analysis findings, we randomly sampled proportions of pyramidal cells in both layers III and V strongly immunostained for the specific metabolic markers, including COX4 and COX1. Both markers showed granular and diffusely spread immunostaining of the neuronal cytoplasm (Figure S5 in supporting information). We initially performed p/a of staining analysis for each metabolic marker. Although these measures revealed trends in mPFC neuronal changes, we found this method to yield inconsistent results across both cortical layers with considerable variances within each disorder (Figure S6 in supporting information). In contrast, we found determining neuronal counts to be more consistent and produced reliable results; further analysis was focused on this method to determine densities in the two‐dimensional plane.

**FIGURE 5:**
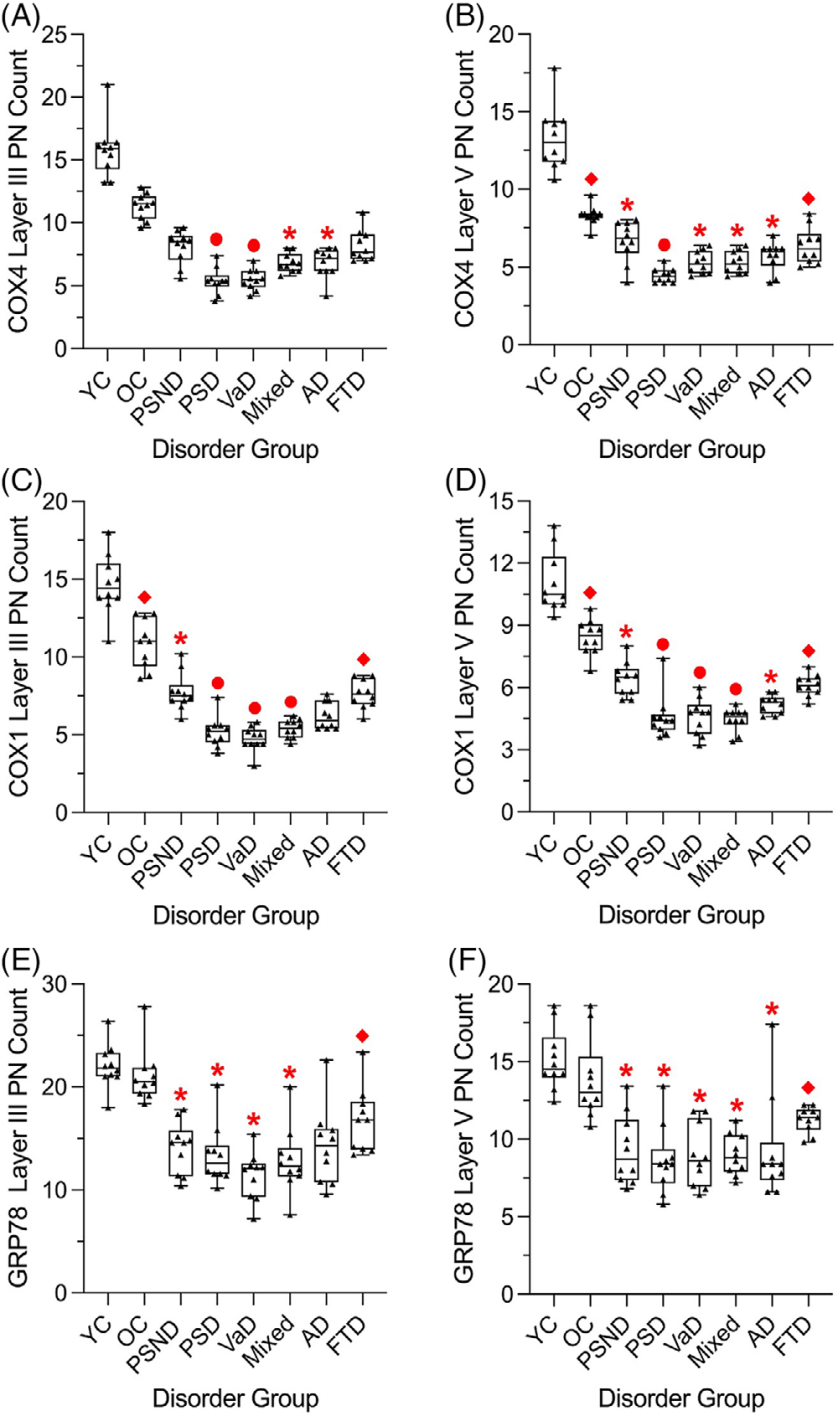
Densitometric analysis of the mPFC neurons with metabolic markers in cortical layers III and V. Box plots show the interquartile range extending to minimum and maximum values as mean pyramidal neuron counts for COX4 (A and B), COX1 (C and D), and GRP78 (E and F) across all disorder groups. Widespread reductions were matched closely with prior three‐dimensional stereology neuronal findings. * significance versus OC, ⬥ differences versus only YC subjects, and • differences versus both OC and PSND cases (*p* ≤ 0.05). Raw data points are shown as triangles. AD, Alzheimer's disease; COX1, cytochrome *c* oxidase subunit 1; COX4, cytochrome *c* oxidase subunit 4; FTD, frontotemporal dementia; GRP78, 78 kDa glucose‐regulated protein; Mixed, mixed dementia; mPFC, medial prefrontal cortex; OC, older controls; PN, pyramidal neuron; PSD, post‐stroke dementia; PSND, post‐stroke non‐dementia; VaD, vascular dementia; YC, younger controls.

We noted reduced COX4 neuronal counts in both cortical layers for all age‐matched dementia groups (*p* < 0.001) and PSND individuals (*p* = 0.03) relative to OC (Figures 5A,B). Both PSD and VaD layer III COX4 neuronal counts were also lower relative to PSND or FTD (*p* < 0.001). PSD exhibited the greatest reduction in layer V COX4 neurons, compared to PSND (*p* < 0.001) and FTD (*p* = 0.006), while OC or FTD were additionally lower for layer V COX4 neuronal counts relative to YC (*p* < 0.001).

We further found widespread reductions in COX1 neuronal counts in PSND as well as all dementia groups relative to OC within both cortical layers (*p* < 0.001; Figures 5C,D). PSD, VaD, and mixed dementia groups exhibited lower COX1 neuronal counts compared to PSND within both cortical layers (*p* < 0.001). COX1 neurons in FTD cases were also reduced relative to YC (*p* < 0.001), whereas the reduction in OC individuals compared to YC (*p* < 0.001) further indicated an aging‐associated effect.

GRP78‐positive neuronal counts in layer III were reduced in almost all dementia groups, including PSD (*p* = 0.008), VaD (*p* < 0.001), and mixed dementia (*p* = 0.003; Figure 5E). Similar trends were exhibited in GRP78 neuronal counts in layer V (Figure 5F) in PSND (*p* < 0.001), PSD (*p* < 0.001), VaD (*p* < 0.001), mixed dementia (*p* < 0.001), and AD (*p* = 0.02) compared to the OC group.

### 3.7 Colocalization of COX4, COX1, and GRP78 metabolic markers

We performed colocalization analysis to detect differential changes and interactions between the metabolic markers within mPFC pyramidal neurons in the most severely affected vascular pathology–related dementias (Figure S7 in supporting information). We found upon visual inspection of five images per case, the mean colocalizations of COX1/COX4 markers were greater than COX4/GRP78 or COX1/GRP78 (*p* < 0.001), while still generally close (78%–85%) in both layers across the PSD, VaD, or mixed dementia subjects. Conversely, the mean frequency of COX1/COX4 colocalized neurons within PSD and mixed dementia cases was consistently found to be reduced by ≈ 8% relative to OC subjects. The mean frequency of COX4/GRP78 or COX1/GRP78 colocalized neurons in both cortical layers across PSD or mixed dementia immunofluorescence images was found to be lower by ≈ 12% compared to the OC subjects (Figure S7).

### 3.8 Association of COX4 and COX1 positive neurons with cognition and aging

We found that COX4 neuronal counts in both layers III and V were positively correlated with MMSE (*r* = 0.556, *p* = 0.011, layer III; *r* = 0.574, *p* = 0.008, layer V) and CAMCOG (*r* = 0.522, *p* = 0.018, layer III; *r* = 0.490, *p* = 0.028, layer V) scores in post‐stroke subjects (Figure S8 in supporting information). COX4 layer III neuronal counts in PSD cases further displayed a positive relationship to PSD frontal vascular pathology scores (*r* = 0.810, *p* = 0.007; Figure S8B).

Similarly, COX1 neuronal counts also exhibited positive correlations in layer III for post‐stroke survivors with MMSE (*r* = 0.628, *p* = 0.003; Figure S8C) and CAMCOG (*r* = 0.622, *p* = 0.003) scores, which was found again for MMSE (*r* = 0.533, *p* = 0.016) and CAMCOG (*r* = 0.493, *p* = 0.027) within layer V. Several other cognitive sub‐scores were positively associated again with COX4 and COX1 neuronal counts including executive function, attention or calculation, as well as orientation and total memory scores. Consistent with the three‐dimensional stereology results (Figure 2), additional analyses as a function of age showed that total COX4, COX1, and GRP78‐positive neuronal counts across both cortical layers were negatively correlated with age for each metabolic marker. For example, COX4 and COX1 appeared to be more affected with age than GRP78 (*r* = −0.235, *p* = 0.036; GRP78; Figure S8D–F).

## 4 DISCUSSION

**FIGURE 6:**
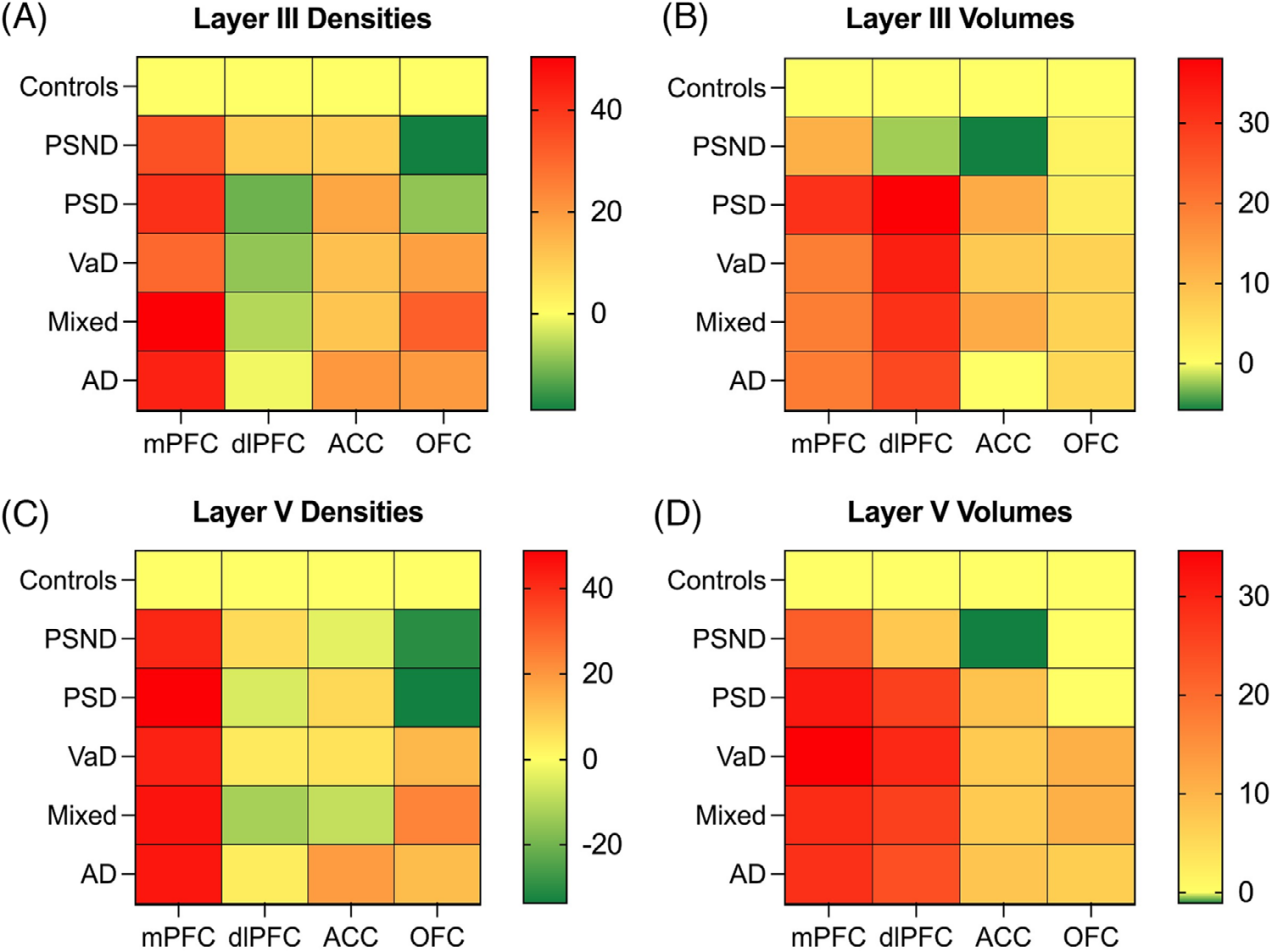
Heat maps illustrating the neuronal changes localized across different prefrontal subregions. Each heat map displays the percent change in neuronal densities (A and C) or volumes (B and D) in both cortical layers for each disorder group relative to baseline OC (yellow). The mPFC appears most vulnerable to deficits by consistently exhibiting large neuronal losses in all assessed groups. Similar differential effects between mPFC and dlPFC were also evident in FTD cases (see the Discussion). Red designates the most severe changes, whereas green indicates higher values relative to the control baseline level. Data were derived from current analysis and Foster et al.^12^ ACC, anterior cingulate cortex; AD, Alzheimer's disease; dlPFC, dorsolateral prefrontal cortex; FTD, frontotemporal dementia; mPFC, medial prefrontal cortex; Mixed, mixed dementia; OC, older controls; OFC, orbitofrontal cortex; PSD, post‐stroke dementia; PSND, post‐stroke non‐dementia; VaD, vascular dementia.

We have provided novel evidence that pyramidal neurons localized in the mPFC undergo widespread morphological changes across common aging‐associated dementias. We found markedly reduced pyramidal neuron densities and volumes in VaD as well as AD and mixed groups by ≈ 37% to 45% in both neocortical layers III and V relative to similarly aged controls. While stroke injury impacts remotely on neuronal integrity as indicated by changes in PSND subjects, our observations suggest neuronal loss is more pivotal than apparent soma shrinkage. This is evident when cognitive dysfunction results directly from neuronal loss.^43^ Prior stereological investigations estimated ≈ 6.5 billion neocortical neurons span the entire prefrontal cortex, with ≈ 280 million pyramidal neurons residing within the mPFC, which is comparable to our estimates given the volume measurements of pyramidal neurons.^44^, ^45^ Taken together with previous findings focused on the dlPFC, anterior cingulate cortex (ACC), and orbitofrontal cortex (OFC) regions within the same brain specimens, we provide strong evidence for considerable mPFC‐specific effects compared to other PFC regions, which was the only prefrontal sub‐region to display significant density changes (Figure 6). It is also noteworthy that similar PFC‐localized differences in pyramidal cell volumes were evident in FTD cases. For instance, neuronal volumes in the mPFC were lower in FTD by 46% to 51% (current study), yet in the dlPFC by only 3% to 16%.^46^

In contrast to neuronal atrophy in the dlPFC,^14^ our new findings imply the dorsal aspects of the mPFC are more vulnerable to pathological processes that cause dementia. Perhaps the mPFC undergoes changes at an earlier stage of prefrontal disease progression, as also conveyed by neuroimaging studies in autosomal dominant AD and mild cognitive impairment.^47^, ^48^ The earliest incidence of functional disconnectivity is involved in mPFC projection areas critical to default mode network cognitive function.^49^ A further important finding was that mPFC layer V neurons may have enhanced susceptibility to morphometric changes, given that all dementia groups exhibited reduced densities and volumes within layer V. This likely resulted from the relatively greater neuroanatomic distances neuronal projections must traverse to subcortical areas.^34^ Hence, aberrations within pyramidal neurons could disrupt mPFC connections to critical subcortical regions such as the hippocampus, leading to cognitive dysfunction.^12^ Although we did not detect differences in neurofibrillary tau pathology burden across vascular‐related dementias, soluble species of tau and amyloid beta (Aβ) have been demonstrated to emerge at earlier Braak stages of progression and precede fibrillar formation.^50^ There is a possibility that soluble species contributed to toxic neurodegenerative effects during early disease stages within the mPFC.

The positive correlations observed within our interlaminar correlation analysis for mPFC neuronal densities suggest neuronal integrity between layers III and V is widespread. We found that neurons within all vascular disease subjects, as well as the AD, mixed, and FTD groups, were affected without extensive evidence of AD‐type neuritic or neurofibrillary pathology. This observation corroborates our previous findings in dlPFC pyramidal neurons,^14^ and supports a vascular etiology for neuronal changes with widespread metabolic deficiencies in these subjects. This reasoning is not only consistent with the presence of cerebral small vessel disease co‐occurring in > 50% of AD‐type dementia patients, but also that microbleed–Aβ interactions have been shown to modify tau accumulation during early stages of AD.^51^, ^52^

Clinical cognitive effects were further emphasized through correlations with MMSE and CAMCOG cognitive scores, as well as executive function deficits in post‐stroke subjects, suggesting that those with worse cognitive function exhibited greater neuronal loss, possibly resulting from impaired axon conduction.^16^ Prior neuroimaging studies showed episodic memory deficits were underpinned by mPFC modulation of hippocampal networks within amnestic mild cognitively impaired individuals. This provides evidence for the mPFC as an early pathogenic substrate of cognitive dysfunction^53^ and is consistent with our previous findings in the hippocampal formation in PSD, VaD, and AD subjects.^37^

In the FTD group, we unexpectedly observed higher neuronal densities yet the largest volume reductions, which negated our initial hypothesis of reduced densities compared to controls. Prior work^54^ using similar blinded stereological methods and Nissl‐stained sections found an ≈ 45% decrease for frontal neuronal densities (cells/mm^3^) in FTD relative to similarly aged controls. However, this study estimated within a combined frontal area averaged across mid‐frontal cortex and OFC regions, and did not discriminate the specific neuronal subtype counted, which is critically important, as indicated by another study investigating the ACC.^54^, ^55^ It therefore appears that an inversely related packing effect has occurred, as implicated by FTD having the smallest mean neuronal volumes. The smaller neurons may have clustered together in the same space, resulting in high densities counted relative to controls.^56^ Because only the FTD group displayed reduced mPFC cortical thickness, it suggests there may be a unique disease‐specific process involved, perhaps resulting from reduced dendritic arborization and early connectivity alterations in frontal–subcortical networks, as emphasized within presymptomatic *MAPT* mutation FTD studies.^57^, ^58^ This finding may also be uniquely related to the high rates of neuropsychiatric symptoms such as apathy consistently observed in FTD patients, particularly since neuroimaging studies previously related such behavioral changes to mPFC‐specific gray matter atrophy.^59^, ^60^, ^61^ Transactive response DNA binding protein 43 kDa (TDP‐43) pathological burden may further contribute toward the exclusive effects within FTD subjects, as type two TDP‐43 sub‐types have previously exhibited distinct atrophy patterns involving the mPFC.^62^ However, only a small subset of PSP cases, which predominantly comprised the FTD group, have reported TDP‐43 intracellular inclusions subsequently spreading to prefrontal cortices, and most of our assessed cases rather displayed tauopathies, negating their potential involvement.^63^

The assessment of both COX4 and COX1 mitochondrial markers in only a fraction of the total layer III or V neurons replicated our three‐dimensional stereology findings of reduced pyramidal neurons across almost all dementia groups, particularly within layer V. Intriguingly, PSD cases had the lowest neuronal counts compared to PSND. This agrees with prior transcriptomic results in frontal pyramidal neurons and may indicate that widespread mitochondrial dysfunction is related to remote vascular effects as a pre‐cell death state in those who develop dementia.^16^ Mitochondrial dysfunction is further implicated in other chronic conditions, as demonstrated by the depletion of COX1‐positive neurons in Parkinson's disease models, GABAergic interneurons in mitochondrial disease, and cholinergic neurons in Lewy body dementia.^21^, ^64^, ^65^, ^66^ However, we found that COX1‐positive neurons were minimally affected (≈ 8% lower) compared to COX4 across the dementias. These discrepancies may stem from differences in using intracellular optical density measurements corrected to porin expression, along with the specific neuronal subtype and brain regions examined.^21^, ^66^ These metabolic marker–related cell changes implicate alterations in cerebral perfusion and, therefore, potentially gliovascular changes in frontal white matter observed within PSD.^67^

One of our most profound observations relates to age. Total neuronal densities across both cortical layers were lower with age, and were further replicated for all three metabolic marker measurements. Equally, mPFC cellular atrophy may be more vulnerable to the initial aging effects regardless of underlying disease etiology within control subjects. These findings were found to be generally consistent within prefrontal cortical regions and in line with von Economo's^42^ reported estimations of neuronal change. Therefore suggesting aging itself aberrantly impacts the mPFC at a localized pyramidal neuronal level, with such neurodegenerative changes perhaps beginning as early as middle age, as was found from patch clamp recordings in non‐human primates.^68^ This was consistent with prior findings of mPFC region–specific susceptibility to aging within cognitive behavioral and network‐based studies.^12^, ^69^ Further emphasis upon targeting pyramidal neurons at an earlier stage, when most neuronal loss appears to arise during the 60 to 69 years decade, may be crucial in preventing downstream pathological sequelae.^14^

Our study explored a wide range of dementia disorders and is strengthened by the fact that all tissue sections were collected, processed, and analyzed in a standardized way to ensure accurate and valid comparisons could be made with minimal differential tissue effects. We would have ideally estimated total neuronal numbers, but quantifying the whole mPFC volume from its demarcated brain regions accurately was not possible, so neuronal density measurements were used instead.^70^ We recognize that using densities as a measure may still have several limitations, which stem from the inherent challenges of stereological analysis upon human *post mortem* tissue.^71^ We did not undertake any immunoblotting analysis of the COX1 and COX4 proteins, as this seemed unnecessary in light of the robust agreement between the stereological results and sampling of marker‐positive neurons using immunohistochemistry.

In conclusion, we have demonstrated abundant mPFC‐specific pathological changes in pyramidal neurons in PSD and other dementias in the general absence of localized neurodegenerative pathology or ischemic infarction. The highly localized neuronal loss and atrophy changes were also associated with cognitive decline and aging effects. We further found an underlying mechanistic effect of reduced mPFC pyramidal neurons immunostained for COX4, COX1, and GRP78, which likely interact at mitochondrial‐associated membranes and become depleted in response to ischemic stress. Our findings collectively emphasize the importance of protecting vascular integrity by preventing cellular metabolic pathways from becoming dysregulated across dementias, potentially through mitochondrially targeted therapies such as antioxidant supplementation to restore neuronal function.^72^ Further work would be required to explore the differential status of connections by using synaptic markers and their spatiotemporal interactions with mPFC pyramidal neurons across aging‐related dementias.

## CONFLICT OF INTEREST STATEMENT

The authors have no disclosures or conflicts of interest in relation to this manuscript. The data that support the findings of this study are available on request from the corresponding author. The data are not publicly available due to privacy or ethical restrictions. Author disclosures are available in the Supporting Information.

## CONSENT STATEMENT

Ethical approvals were granted by local research ethics committees of the Newcastle upon Tyne Foundation Hospitals Trust. Permission for use of brains for *post mortem* research was also granted by consent according to the Declaration of Helsinki from the next‐of‐kin or family. All the brain tissue was retained in and obtained from the Newcastle Brain Tissue Resource.

## Supporting information



Supporting Information

Supporting Information
