# Supplementary material for: High vulnerability of medial prefrontal pyramidal neurons in post‐stroke, vascular, Alzheimer's disease, and aging‐related dementias
Source: Alzheimers Dement. 2026 Feb 12;22(2):e71151. doi: 10.1002/alz.71151 (PMC12897552; doi:10.1002/alz.71151)
Supplement: Supplementary file 1 — Supporting Information [file ALZ-22-e71151-s002.pdf]

Supplementary Materials: **TABLE S1.** Layer III and V neuronal densities or volumes for all disorder groups.

Disorder Group	Layer III Neuronal Density		Layer V Neuronal Density		Layer III Neuronal Volume		Layer V Neuronal Volume	
	Mean (±SEM)	*Median (Range)*	*Mean (±SEM)*	*Median (Range)*	*Mean (±SEM)*	*Median (Range)*	*Mean (±SEM)*	*Median (Range)*
YC (n = 10)	33520 (±1328)	32400 (±13600)	25573 (±724.8)	25200 (6667)	2207 (±92.6)	2369 (756.3)	2601 (±66.9)	2629 (606.8)
OC (n = 11)	31054 (±2417)	30800 (27067)	25091 (±2212)	24400 (22399)	1778 (±142.3)	1731 (1763)	2278 (±152.3)	2355 (1642)
PSND (n = 12)	20311 (±1705)	20000 (18000)	14678 (±946.7)	15000 (11067)	1565 (±85.1)	1635 (832.4)	1779 (±89.5)	1716 (999.5)
PSD (n = 12)	18250 (±2077)	17600 (25866)	12850 (±784.4)	13333 (9067)	1231 (±79.4)	1189 (771.6)	1561 (±66.8)	1465 (767.7)
VaD (n = 10)	21760 (±2203)	21200 (19467)	14427 (±1068)	13867 (10267)	1434 (±84.6)	1505 (731)	1490 (±67.6)	1508 (613.9)
Mixed (n = 11)	15388 (±1152)	15067 (12133)	13691 (±877.6)	13067 (9067)	1435 (±95.2)	1298 (848.1)	1621 (±88.5)	1512 (1033)
AD (n = 10)	17400 (±1177)	18267 (11867)	13813 (±622.6)	14600 (6533)	1429 (±91.6)	1422 (917.2)	1636 (±113)	1614 (1276)
FTD (n = 15)	40000 (±2701)	37867 (34933)	30498 (±1816)	28933 (23333)	961 (±82.2)	944 (1238)	1115 (±105)	1140 (1171)
Total (n = 91)	25279 (±1159)	22667 (52400)	19259 (±854.7)	16533 (34800)	1473 (±49.2)	1447 (2301)	1725 (±57.5)	1642 (2567)

Note: Data consists of mean or median estimates of pyramidal cell neuronal densities and volumes for each cortical layer and disorder group, with the sample size of subjects denoted by *n* and the standard error mean (SEM) or range displayed in parentheses. The total row represents the mean or median values for all disorder group values combined. Abbreviations: AD, Alzheimer's disease; FTD, frontotemporal dementia; OC, older controls; PSD, post-stroke dementia; PSND, post-stroke non-dementia; VaD, vascular dementia; YC, younger controls.

TABLE S2. Interlaminar neuronal density and volume correlations.

	YC	OC	PSND	PSD	VaD	Mixed	AD	FTD
Densities (cells/mm^3) Layer III vs. Layer V *r* (*P* value)	0.658 (0.039)	**0.504 n.s.**	0.739 (0.006)	0.600 (0.039)	0.670 (0.034)	0.631 (0.037)	0.814 (0.004)	0.831 (<0.001)
Volumes (μm^3) Layer III vs. Layer V *r* (*P* value)	**0.423 n.s.**	0.631 (0.037)	**0.102 n.s.**	**0.337 n.s.**	**0.200 n.s.**	0.751 (0.008)	0.662 (0.037)	0.813 (<0.001)

Note: For interlaminar neuronal comparisons, the data values show correlation coefficients (r) from Pearson's correlation analysis, followed by *P* values in parentheses. Interlaminar neuronal densities (cells/mm^3) were not significantly correlated only for the OC group (in bold) suggesting a widespread effect of neuronal loss not limited to specific cortical layers appears to exist throughout all dementia groups. Whereas interlaminar neuronal volumes (μm^3) were not significantly correlated in PSND, PSD, VaD plus YC (in bold) implicating potential differential cellular changes with ageing or disease between the cortical layers III and V. Abbreviations: AD, Alzheimer's disease; FTD, frontotemporal dementia; mPFC, medial prefrontal cortex; n.s., non-significant ($P > 0.05$); OC, older controls; PSD, post-stroke dementia; PSND, post-stroke non-dementia; VaD, vascular dementia; YC, younger controls.

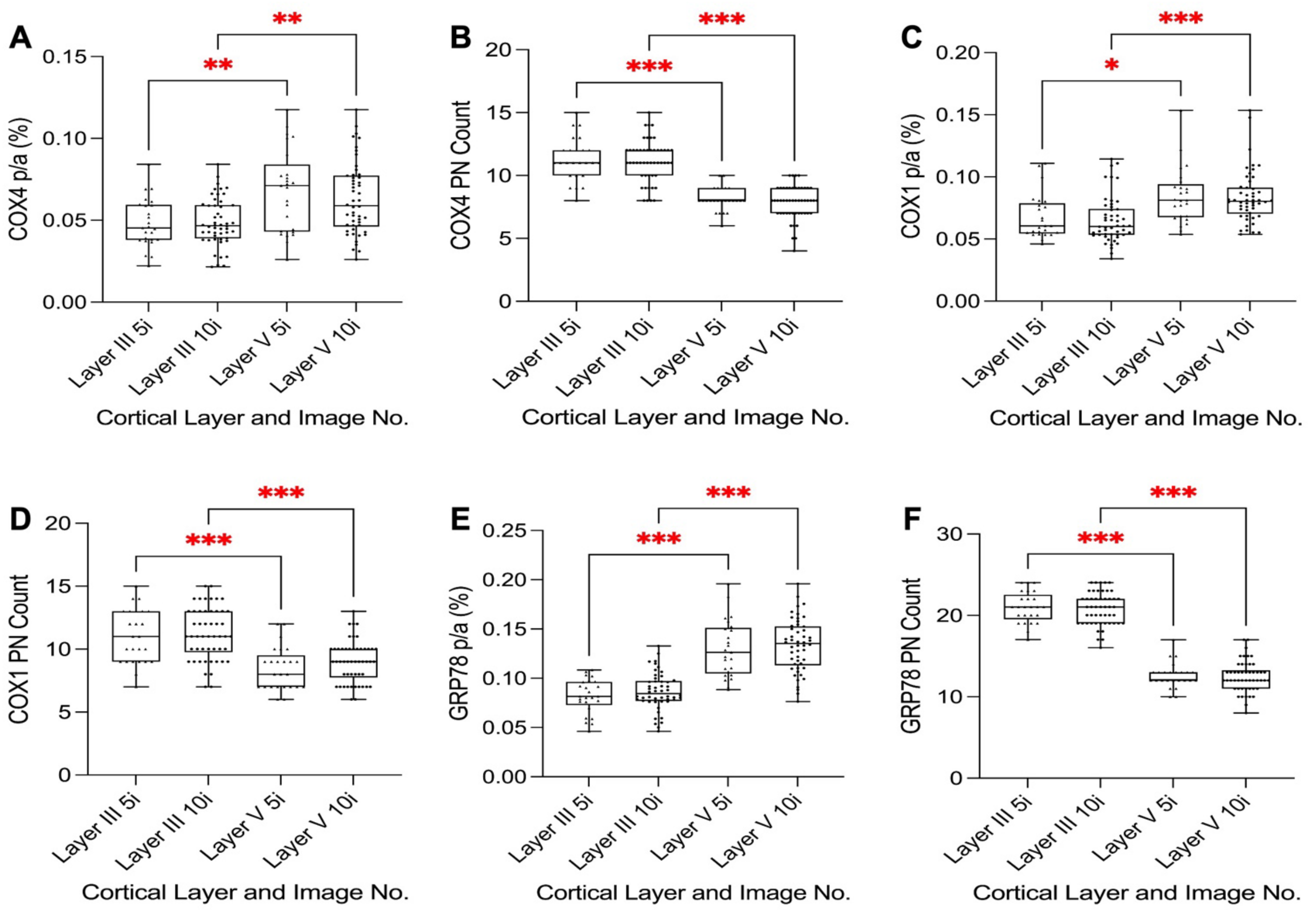

FIGURE S1. Comparison of COX4, COX1 and GRP78 p/a of staining or neuronal counts. Box plots represent the interquartile range extending to minimum and maximum values. No significant differences ($P > 0.05$) for capturing either 5 or 10 images per cortical layer were found within five control cases for both % area (A, C and E) or pyramidal neuron count (B, D or F) mean (± SEM) values. Significant differences were only suggested between the cortical layers for both p/a and neuronal count across all COX4 (A and B), COX1 (C and D) or GRP78 (E and F) metabolic markers. For significant differences, * $P < 0.05$, ** $P < 0.01$ and *** $P < 0.001$. Raw data points are displayed as triangles for 5 images or circles for 10 images. Abbreviations: 5i, five images; 10i, ten images; No., number; p/a, per area percentage; PN, pyramidal neuron.

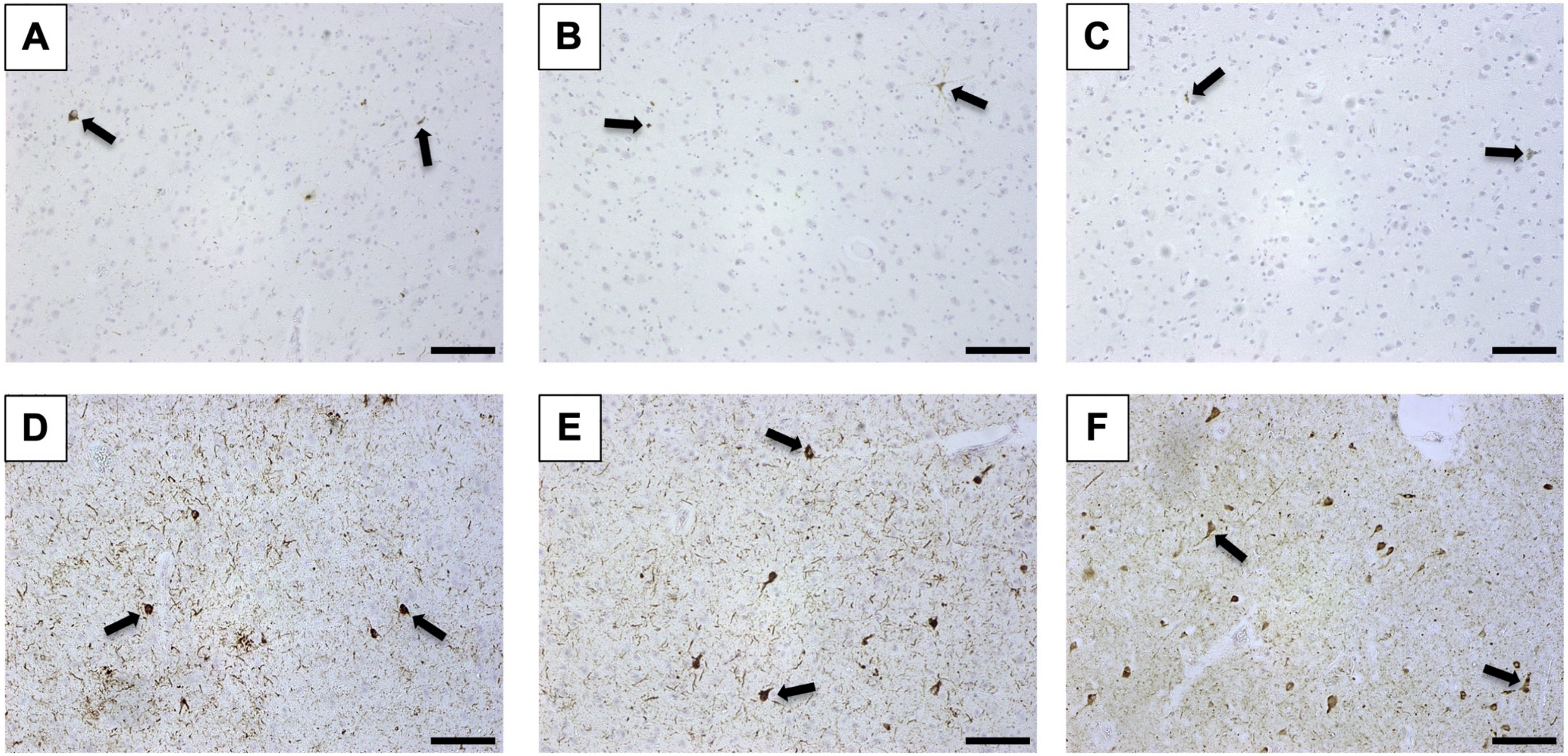

FIGURE S2. Brightfield images of hyperphosphorylated tau tangle pathology in dementia groups. Representative images from mPFC layer V shows extent of neocortical neurofibrillary pathology. Negligible AT8 immunostaining was evident in PSND (A), PSD (B) and vascular dementia (C) subjects, whereas extensive hyperphosphorylated tau tangles were observed for mixed dementia (D), AD (E) and FTD (F) cases. Arrows depict positive AT8 immunoreactivity. Scale bar = 100µm. Abbreviations: AD, Alzheimer's disease; FTD, frontotemporal dementia; mPFC, medial prefrontal cortex; PSD, post-stroke dementia; PSND, post-stroke non-dementia; VaD, vascular dementia.

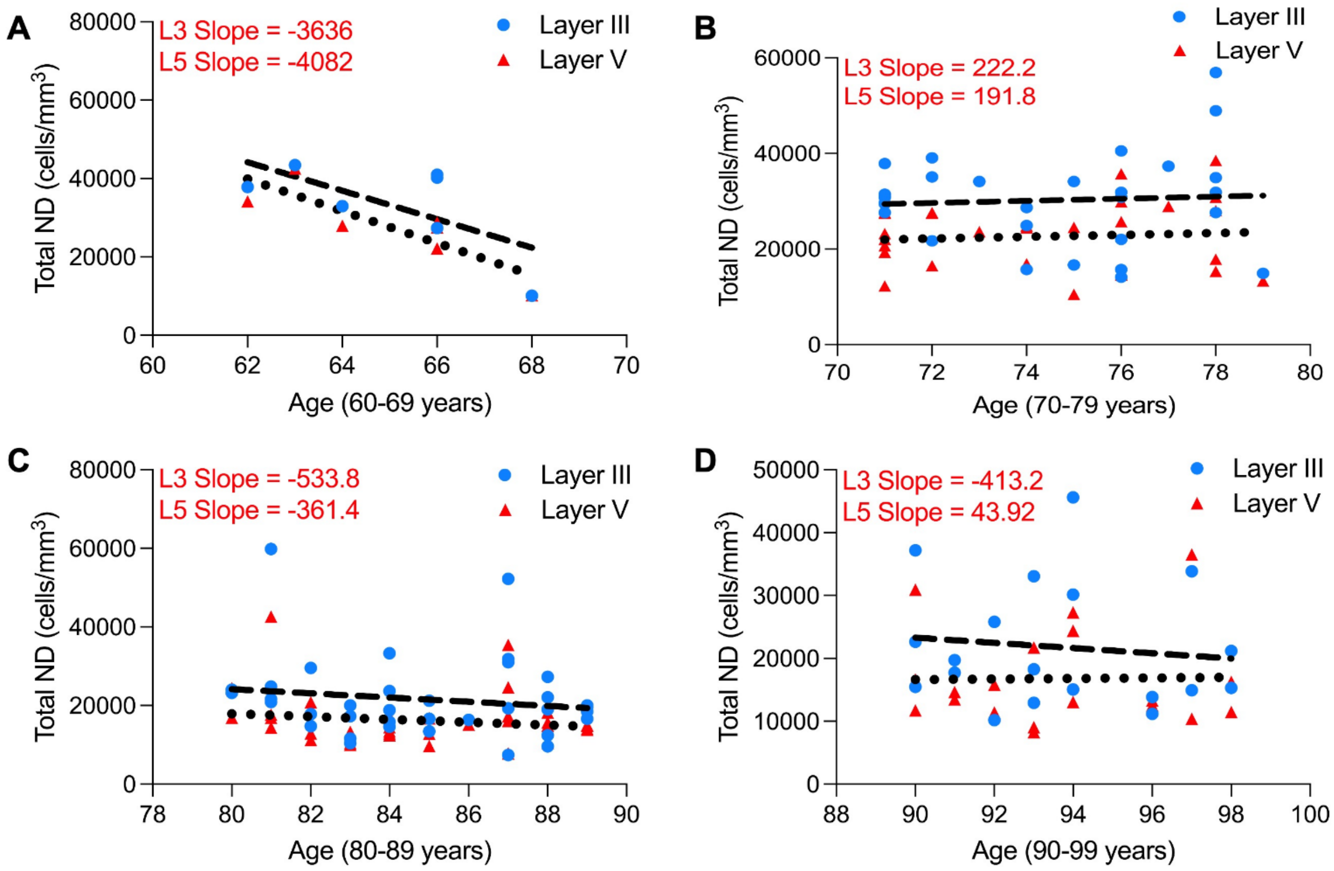

FIGURE S3. Associations of total neuronal density with age stratified into decades. Linear regression showing total neuronal densities (ND) in layers III (L3) and V (L5) plotted against age (years) separated into decades: 60-69 (A), 70-79 (B), 80-89 (C) or 90-99 (D). The slope of each simple linear regression line was then measured for each decade and Pearson's correlation coefficient implicated that the 60-69 decade (A) influenced most to the overall negative relationship of lower densities with increasing age in layer V (r = -0.851; P = 0.015). Yet, it is important to note that this trend does have the implicit caveat of only encompassing seven data points for cases aged 60-69 years. Dashed or dotted lines-of-best-fit indicate either layer III or layer V, whilst symbols show raw data points for all linear regression curves, with significance at $P \leq 0.05$.

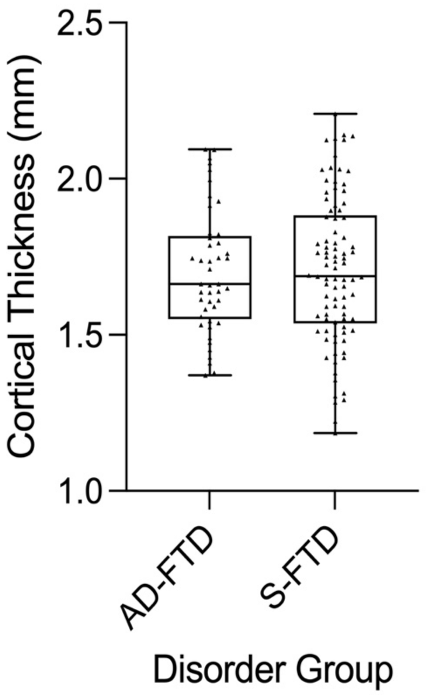

FIGURE S4. Morphological cortical thickness estimations for the FTD group. The box plot shows the interquartile range extending to minimum and maximum values, with no differences ($P > 0.05$) apparent between FTD sub-groups of familial autosomal dominant (AD-FTD) or sporadic (S-FTD) four-repeat tauopathy in mean cortical thickness measurements (± SEM). They showed almost identical mean cortical thickness lengths of 1.702mm (±0.029 SEM) or 1.707mm (±0.025 SEM), respectively. Raw data points are shown as triangles.

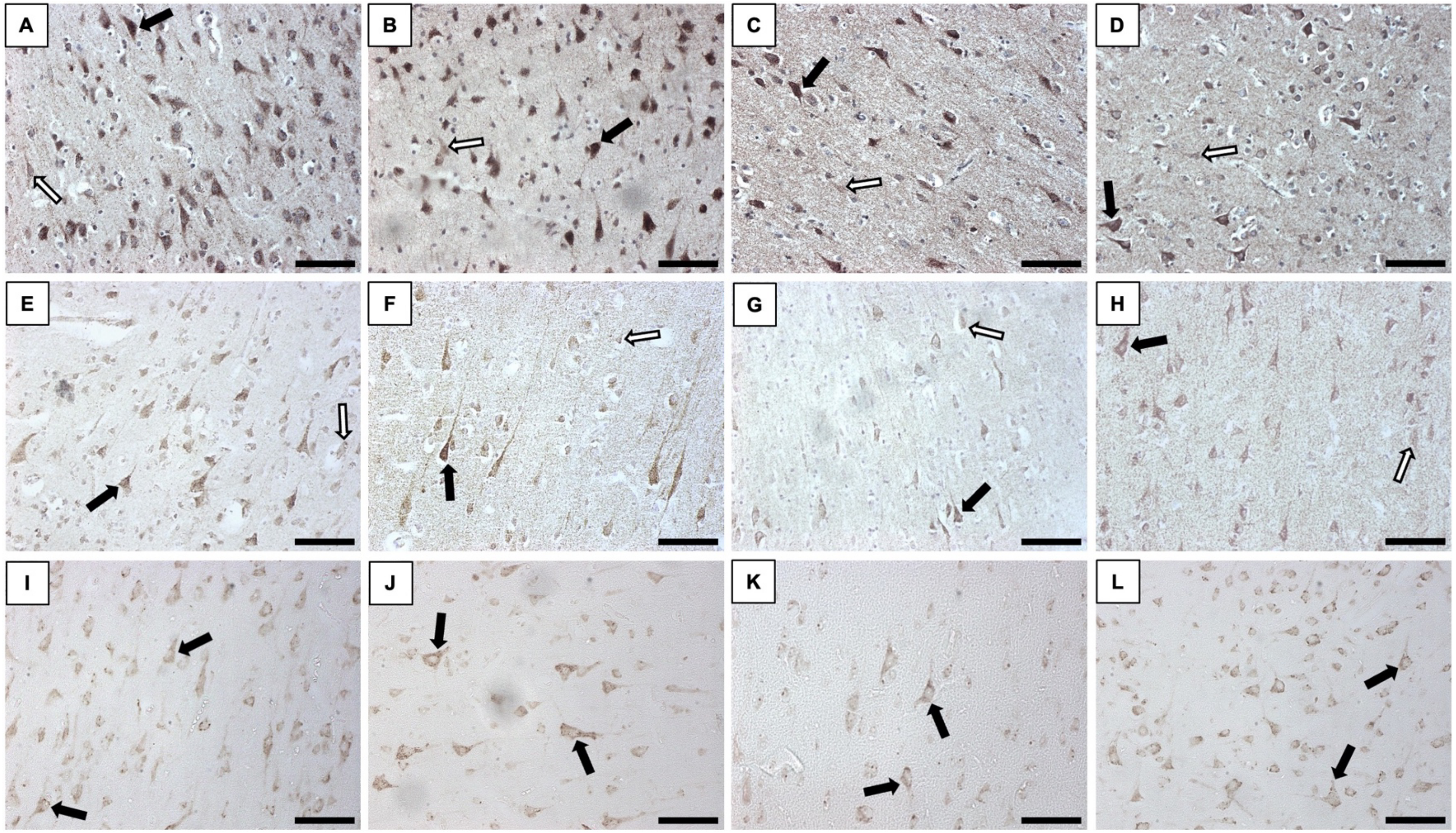

FIGURE S5. Immunocytochemical localisation of COX4, COX1 or GRP78 metabolic markers in mPFC pyramidal neurons. Representative images of COX4 (A-D), COX1 (E-H) and GRP78 (I-L) immunostaining in layer V in OC (A, E and I), PSND (B, F and J), PSD (C, G and K) and vascular dementia (D, H and L) subjects. Open arrows indicate lighter stained cells (not counted), whereas solid arrows represent intensely positive stained neurons. Scale bar = 50µm.

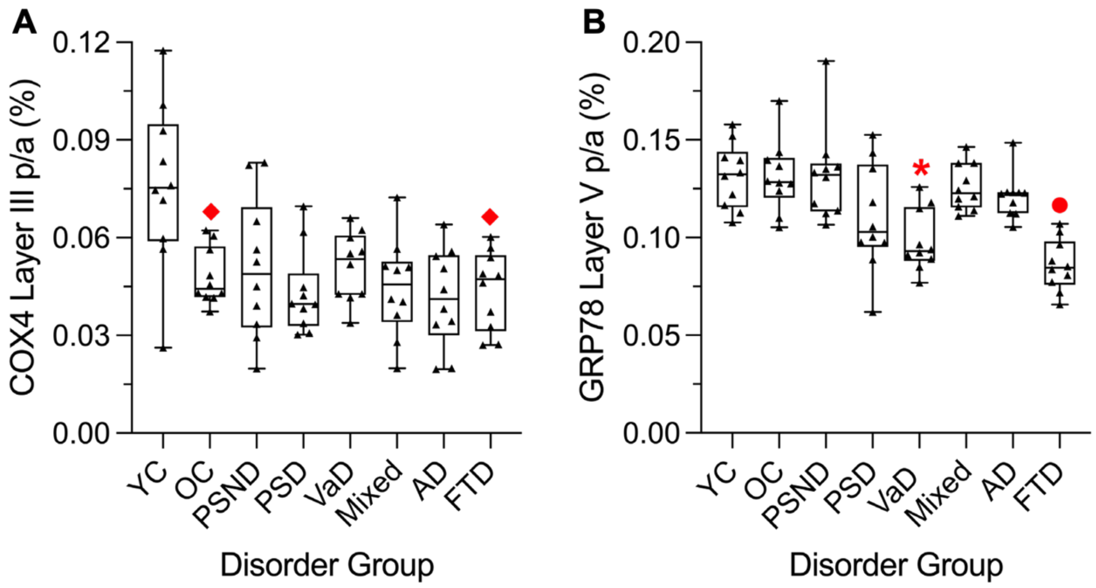

FIGURE S6. Densitometry p/a of staining for COX4 and GRP78 metabolic markers. Box plot graphs display the interquartile range extending to minimum and maximum values, with a large degree of variability for the mean p/a of staining values (± SEM) for COX4 layer III (A) or GRP78 layer V (B) across all assessed disorder groups. (A) For COX4 layer III p/a values, only the YC subjects compared to OC (*P* = 0.005) or FTD (*P* < 0.001) groups were lowered across all disorder groups. (B) Whereas, lower GRP78 layer V p/a was only exhibited for OC compared to VaD *(P* = 0.003*),* as well as FTD relative to YC (*P* < 0.001) or PSND (*P* = 0.002) cases. FTD compared to mixed dementia (*P* < 0.001) or AD *(P* = 0.04)*,* plus VaD relative to mixed dementia (*P* = 0.02) were further reduced. * different mean values vs. OC, ◆ differences vs. only YC subjects and ● differences vs. both YC and PSND cases (significance is P ≤ 0.05). Raw data points are illustrated as triangles. Abbreviations: AD, Alzheimer's disease; FTD, frontotemporal dementia; Mixed, mixed dementia; OC, older controls; PSD, post-stroke dementia; PSND, post-stroke non-dementia; p/a, per area percentage; VaD, vascular dementia; YC, younger controls.

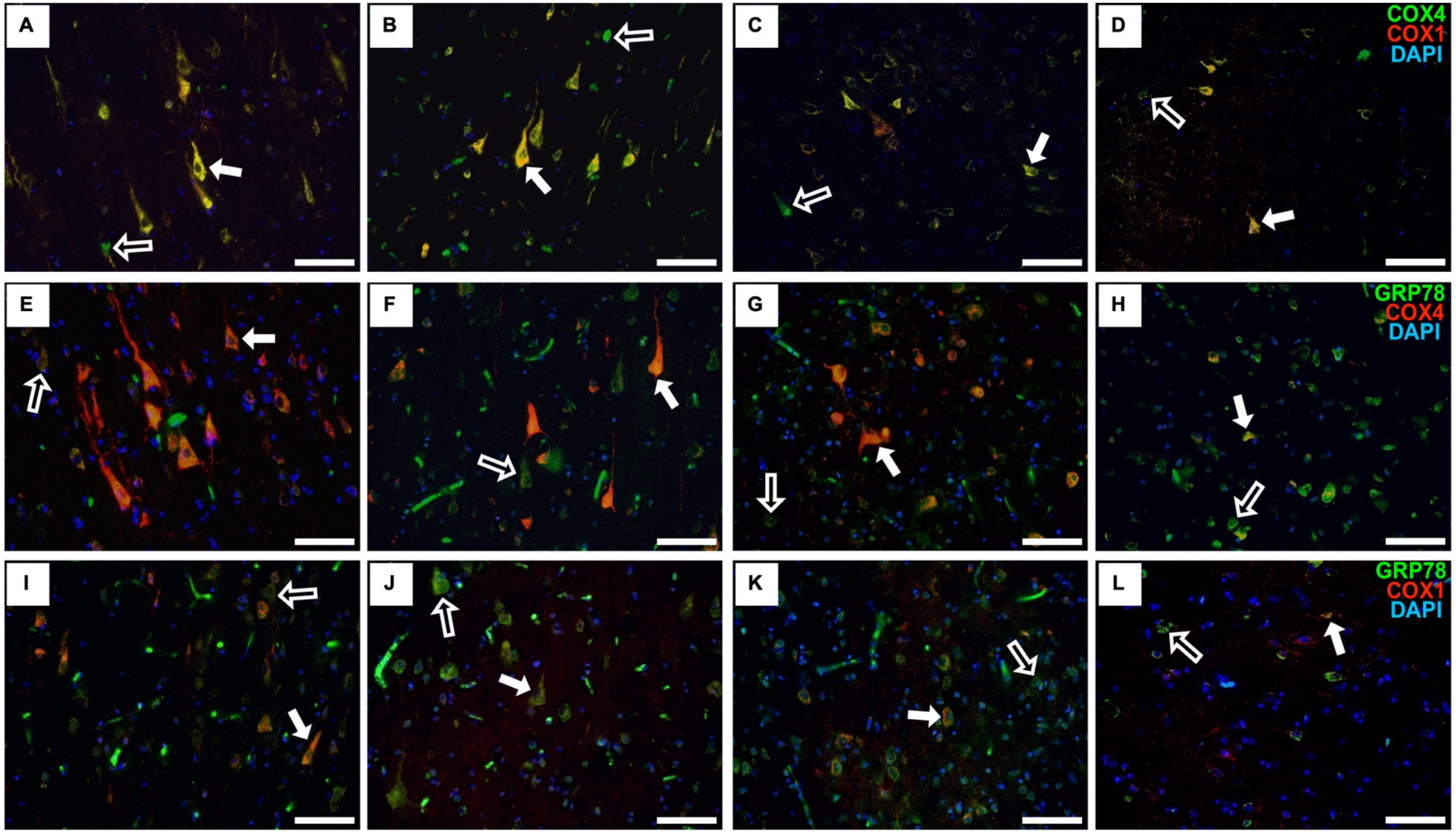

FIGURE S7. Immunolocalisation of different metabolic neuronal markers in cortical layer V. (A-D) COX4 (green) and COX1 (red) exhibit almost complete overlapping of these two markers. (E-H) GRP78 (green) and COX4 (red) immunofluorescent staining with nuclei in blue (DAPI). (I-L) GRP78 (green) and COX1 (red) also show co-localisation of these markers. Four separate disorder groups were selected for comparison: OC (A, E and I), PSD (B, F and J), vascular dementia (C, G and K) and mixed dementia (D, H and L). Solid arrows show co-localised neurons, whereas open arrows highlight neurons without co-localisation (yellow) signal. Scale bar = 50µm.

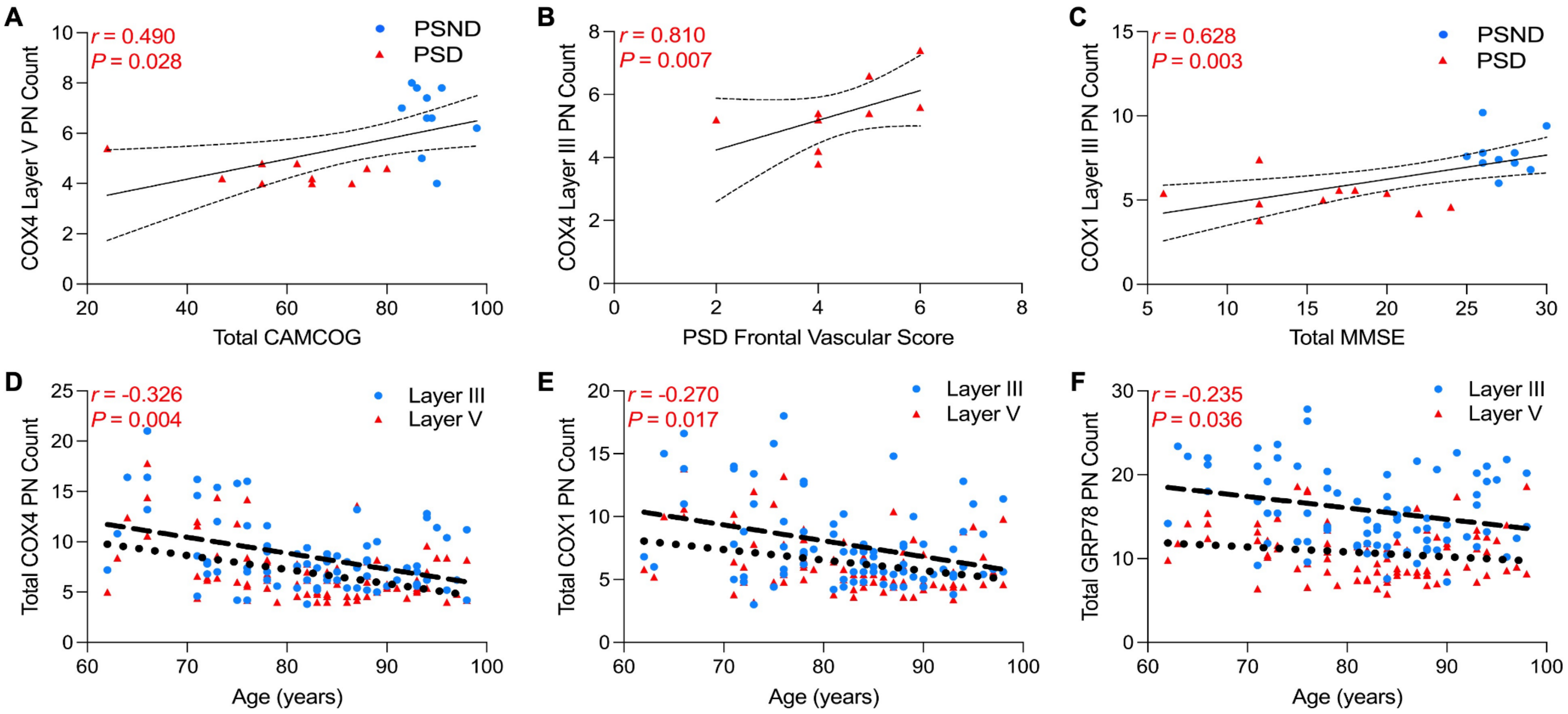

FIGURE S8. Metabolic marker associations with the clinical variables of cognition, vascular pathology or age. Positive correlation curves show a linear relationship between post-stroke cases for cognitive MMSE or CAMCOG scores respectively, in terms of COX4 layer V neuronal count (A) or COX1 layer III neuronal count (C). Linear relationships between frontal vascular score and COX4 layer III neuronal count in PSD subjects (B). The total neuronal counts for COX4 (D), COX1 (E) and GRP78 (F) across both layers III and V declined with age. Dashed lines highlight 95% confidence bands of the regression line (A-C), whilst dashed or dotted lines-of-best-fit represent cortical layers III or V (D-F). Spearman's rank correlation coefficient was utilised due to the non-parametric data. Significance was deemed as $P \leq 0.05$. Abbreviations: CAMCOG, Cambridge Cognition Examination; MMSE, Mini-Mental State Examination; PSD, post-stroke dementia; PSND, post-stroke non-dementia; PN, pyramidal neuron.
